# Cognitive and MRI trajectories for prediction of Alzheimer’s disease

**DOI:** 10.1038/s41598-020-78095-7

**Published:** 2021-01-22

**Authors:** Samaneh A. Mofrad, Astri J. Lundervold, Alexandra Vik, Alexander S. Lundervold

**Affiliations:** 1 Department of Computer Science, Electrical Engineering and Mathematical Sciences, Western Norway University of Applied Sciences, Pb. 7030, Bergen, 5020 Norway; 2 Department of Biological and Medical Psychology, University of Bergen, Bergen, Norway; 3 MMIV, Department of Radiology, Haukeland University Hospital, Bergen, Norway

**Keywords:** Predictive markers, Dementia, Alzheimer's disease, Magnetic resonance imaging, Cognitive ageing

## Abstract

The concept of Mild Cognitive Impairment (MCI) is used to describe the early stages of Alzheimer’s disease (AD), and identification and treatment before further decline is an important clinical task. We selected longitudinal data from the ADNI database to investigate how well normal function (HC, n= 134) vs. conversion to MCI (cMCI, n= 134) and stable MCI (sMCI, n=333) vs. conversion to AD (cAD, n= 333) could be predicted from cognitive tests, and whether the predictions improve by adding information from magnetic resonance imaging (MRI) examinations. Features representing trajectories of change in the selected cognitive and MRI measures were derived from mixed effects models and used to train ensemble machine learning models to classify the pairs of subgroups based on a subset of the data set. Evaluation in an independent test set showed that the predictions for HC vs. cMCI improved substantially when MRI features were added, with an increase in $F_1$-score from 60 to 77%. The $F_1$-scores for sMCI vs. cAD were 77% without and 78% with inclusion of MRI features. The results are in-line with findings showing that cognitive changes tend to manifest themselves several years after the Alzheimer’s disease is well-established in the brain.

## Introduction

Ageing is associated with cognitive changes characterised by phenotypic diversity in both pace and severity. This diversity is a result of the many biological and life-style factors influencing an individual throughout his or her life-time^1,2^. Some individuals preserve their cognitive function into old age, so-called “superagers”^3^, while others experience a decline at a younger age due to a neurodegenerative disease^4^. Along this wide dimension of cognitive function, it becomes difficult to define the fine line between normal and pathological ageing.

Alzheimer’s disease (AD) is a common neurodegenerative disease characterised by a cognitive impairment that gradually worsens over time^5^. A lot of effort has been put into the identification and development of treatment options that can stop this degenerative process at an early stage. Early on, the cognitive symptoms tend to be minor and the condition is referred to as a Mild Cognitive Impairment (MCI)^6^. Not all patients with MCI will develop AD. Although studies have shown that a patient with MCI has up to a tenfold increased risk to develop the disease^4,7^, a subgroup of individuals with MCI are left with a stable condition or may even revert to normal function^8^. The search for predictors of conversion from MCI to AD is therefore an important field of research^6,9^.

Impaired performance on psychometric tests of memory function^10,11^ and on more global measures of cognitive function^9^ have been recognized as early cognitive predictors of AD. However, this impairment tend not to be uncovered until years after the condition is well-established in the brain^12^. This is documented by several previous studies relating early changes in cognitive function to changes in specific regions and structures of the brain, including an expansion of the ventricles and volume loss in the hippocampus and entorhinal cortex^13,14^. A more precise prediction of AD is therefore expected if information from results on cognitive tests are combined with information from magnetic resonance imaging (MRI) of the brain^15,16^.

The present study was motivated by the challenge to predict AD at an early stage of the disease. Based on data available from the Alzheimer’s Disease Neuroimaging Initiative (ADNI) we investigated how well a set of machine learning models could predict conversion from normal function through MCI to AD. In a first set of analyses we defined features characterising longitudinal changes in memory function (Rey Auditory Learning Test (RAVLT))^11^ and in a more global measure of cognitive function (ADAS-Cog-13 (ADAS13))^9,17^. Expecting more precise predictions by including information from MRI examinations^15,16^, we investigated the add-on effect of including morphometric brain measures associated with memory function (entorhinal cortex and hippocampus^14^) and a global measure of cognitive function (the volume of the ventricles as a proxy for a global tissue loss^18^). More specifically, we used a pipeline proposed by Mofrad et al.^19^ based on a combination of mixed effects and machine learning models for analysis of longitudinal data. This approach is useful when faced with a set of subjects with a varying number of scans and test results, examined at different time intervals. This is a common challenge in longitudinal studies, including studies based on the ADNI dataset.

## Materials and methods

### Data set

Data were obtained from the Alzheimer’s Disease Neuroimaging Initiative (ADNI) database (http://adni.loni.usc.edu). The ADNI was launched in 2003 as a public-private partnership, led by Principal Investigator Michael W. Weiner, MD, with an overall goal to validate biomarkers for use in clinical treatment trials for patients with AD. The study was approved by the Institutional Review Boards at each ADNI site (see full list here: http://adni.loni.usc.edu). Informed consent was obtained from all subjects prior to enrollment. All methods were carried out in accordance with relevant guidelines and regulation. The present study was approved by ADNI Publication Committee (ADNI DPC).

In the present study we included subjects from the ADNI dataset defined as cognitively normal (CN) or as patients with an MCI or AD diagnosis. According to the ADNI protocol, MCI was defined if a participant or caregivers reported cognitive problems, if the patient showed impairment on the logical memory-II subtest from Wechsler memory scale-R, a mini-mental state examination score equal to or above 24, and a clinical dementia rating = 0.5. None of the participants with MCI should meet the diagnostic criteria for dementia. AD was diagnosed according to the NINCDS-ADRDA Alzheimer’s Criteria for probable AD (see http://adni.loni.usc.edu/methods/documents for details).

We defined four subgroups from the ADNI sample, with a restriction to subjects with MRI scans at least at two time-points and results on two selected psychometric tests of cognitive function. We labelled subjects as *healthy controls* (HC) if they were classified as CN at all ADNI visits. The subjects who converted from CN to MCI during the observation period were labelled *converted MCI* (cMCI). Subjects who were defined with MCI at all visits were labelled *stable MCI* (sMCI) and those converting from MCI to AD were labelled *converted AD* (cAD) (see Table 1). We balanced the number of subjects in each pair of subgroups, (HC, cMCI) and (sMCI, cAD), controlling for age and gender, and ended up with a total of 934 subjects. See Tables  2 and 3 for details.

**Table 1:** The original ADNI labels and the longitudinal labels used in the present study.

Labels in ADNI and our longitudinal labels		
Labels	Subgroup	Description
ADNI	CN	Cognitively normal at visit
	MCI	Mild cognitive impairment at visit
	Dementia	Alzheimer’s disease at visit
Our study	HC	CN at all visits
	cMCI	Initially CN but later converted to MCI
	sMCI	MCI at all visits
	cAD	Initially MCI but later converted to AD
	sAD$^{\mathrm{a}}$	Dementia at all visits

${}^{\mathrm{a}}$ The sAD subgroup was not included in the present study as we focused on converters.

**Table 2:** Total number of subjects, T1-weighted MR images, and gender distribution within each of the four subgroups. The table also shows the average number of MRI scans (mri) and cognitive tests (cog) per subject, available in each subgroup, and the average time (in years) between the MRI scans and cognitive tests per subgroup.

Information about the subgroups					
Subgroups	$\#$Subjects	$\#$Images	Gender (f/m)	Average $\#$visits (mri/cog)	Average time (mri/cog)
HC	134	642	57/77	4.8/6.0	0.58/0.78
cMCI	134	731	55/79	5.5/7.0	0.62/0.80
sMCI	333	1696	143/190	5.1/6.0	0.48/0.63
cAD	333	1871	130/203	5.6/6.5	0.53/0.62
ALL	934	4904	385/549	5.3/6.3	0.54/0.67

### Cognitive and MRI measures

**Table 3:** Means and standard deviations of age, education level, and scores on the included cognitive tests for each subgroup, given separately for the training and test sets. The information for the converted subgroups (cMCI and cAD) is calculated after removing the measurements from point of conversion and onward. The p-values for pairs of subgroups are presented separately for females and males; $$*$$: p < .05; $$**$$: p < .01; $$***$$: p < .001; −non-significant at 0.05 level.

Variables	Subgroups				p-values
	HC	cMCI	sMCI	cAD	(HC-cMCI)/(sMCI-cAD)
**Age**					
Train (f/m)	77.4±7/77.7±7	75.2±7/77±7	75.2±8/77±7	75.6±8/77.6±7	($$***$$/−)/(− /−)
Test (f/m)	77.2±7/78.3±6	76.6±9/77.2±6	72.6±6/76±7	72.3±8/77.2±7	(−/−)/(−/−)
**Education**					
Train (f/m)	15.1±3/ 17.5±2	16±2/17±2	15.6±3/16.5±3	15.1±3/16.2±3	($$***$$/$$***$$)/($$**$$/−)
Test (f/m)	16.1±3/17.2±3	17±2/15.8±4	13.1±3/15.8±3	15.9±3/16.4±3	($$*$$/$$**$$)/($$***$$/$$*$$)
**RAVLT-Im**					
Train (f/m)	47.4±10/43.8±11	47.3±10/39.6±10	38.7±11/33.2±10	29.4±9/28.2±7	(−/$*$)/($*$/$*$)
Test (f/m)	48.3±8/40.8±8	51.3±14/35.5±7	38.4±12/32.8±10	30.1±10/26.4±6	(−/$$***$$)/($$***$$/$$***$$)
**RAVLT-PF**					
Train (f/m)	30.8±27/36±30	33.1±26/43±27	54.7±33/58.4±32	81.8±28/77.1±27	(−/$$**$$)/($$***$$/$$***$$)
Test (f/m)	31.4±25/35.9±25	33.8±28/48.3±29	51.9±35/56.1±32	81.6±30/81.4±25	(−/$$**$$)/($$***$$/$$***$$)
**ADAS13**					
Train (f/m)	8.2±4/9.9±5	8.6±4/10.9±5	14.3±7/15.3±7	21.8±7/19.7±6	(−/$$**$$)/($$***$$/$$***$$)
Test (f/m)	8.1±4/8.1±3	8.3±5/12.2±3	13.8±8/15.7±6	22.1±7/20.4±6	(−/$$***$$)/($$***$$/$$***$$)

The *RAVLT* was included as a measure of memory function. In this test, the participants are asked to recall words from a list of 15 nouns immediately after each of five learning trials and after a short and a long delay. Two measures known to be sensitive to cognitive changes in patients with AD^11^ were included in the present study: *Immediate recall* (RAVLT-Im): the number of correct responses across the immediate recall of the five learning trials; *percent forgetting* (RAVLT-PF): the score on the fifth learning trial minus the score on the long delayed recall, divided by the score obtained on the fifth learning trial. The lower the scores, the more severe impairment of cognitive function.

The *ADAS13* was included as a global measure of cognitive function. ADAS13 is a test battery developed to assess severity of cognitive impairment associated with AD and includes subtests and clinical evaluations assessing memory function, reasoning, language function, orientation and praxis. The ADAS13 is a modified version of the original ADAS-Cog-11^20^, adding a cancellation task and a delayed free recall task^21^. The higher the scores, the more severe impairment of cognitive function.

We used Freesurfer v.6.0^22^ to derive measures from the T1-weighted MR images, extracting the lateral ventricle volumes, the volumes of the hippocampus and the thickness of the entorhinal cortex in the left and right hemisphere. To reduce the effect of individual and gender differences in brain sizes, the volumes were normalized using a total intracranial volume measure estimated by Freesurfer (eTIV).

**Figure 1:**
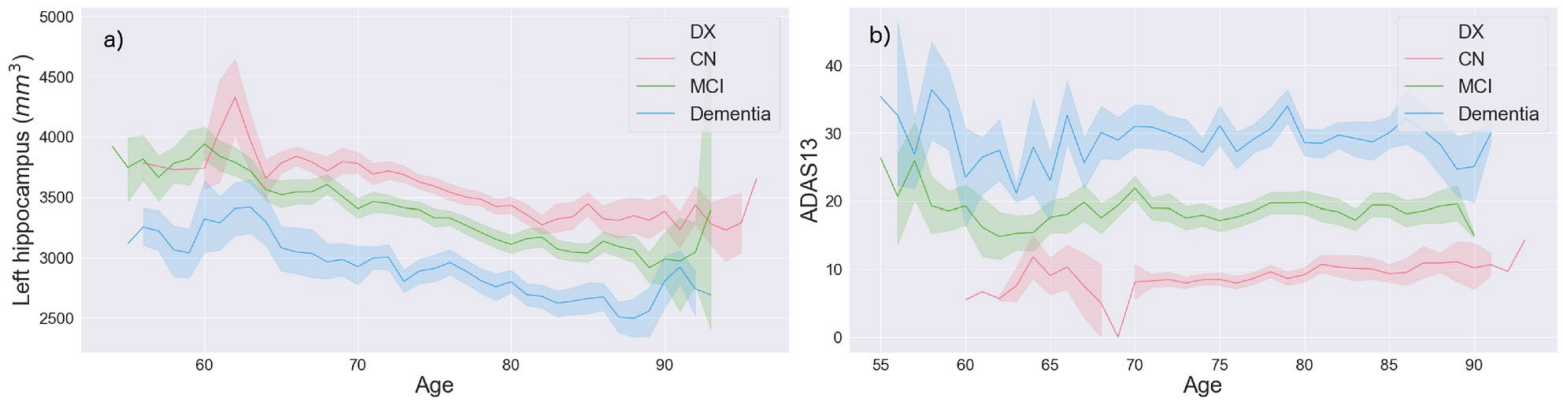
Mean values for (**a**) the volume of the left hippocampus, and (**b**) the ADAS13 score over age, based on the cross-sectional ADNI labels.

**Figure 2:**
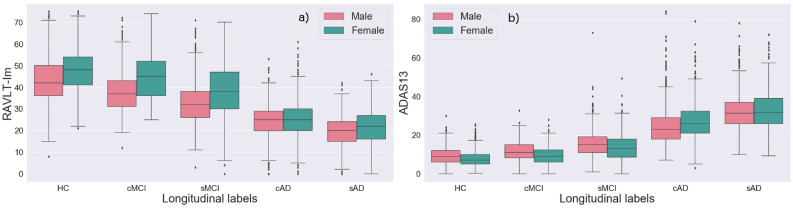
Box plot showing the gender specific results on RAVLT immediate recall and the ADAS13 for each of the longitudinal labels defined for the present study (Table 1).

Figure 1 shows the age-dependent volume changes in the hippocampus (left hemisphere) and ADAS13 test scores across age. The severity of the volume loss and impairment on the ADAS13 are gradually increased from the HC through MCI to AD in the ADNI dataset. Figure 2 illustrates that the more severe scores in patients with AD compared to the other groups are found in both males and females, with a trend towards higher scores (i.e., better results) in females than males on the memory test in the CN and the MCI groups. Means and standard deviations for the RAVLT and the ADAS13 test scores are presented in Table 3.

### Features

To construct subject specific trajectories for each measure we used linear mixed effects models^23,24^, a class of models able to produce regression models from dependent variables^25^. Our models are based on the one presented in^24^ and similar to the ones employed in our previous works^19,26^. As the ventricles show quadratic cohort behaviour (Fig. 5), likely caused by the accumulation of cerebrospinal fluid due to atrophy in multiple brain regions, we used linear mixed effects models both with and without a quadratic covariate term:

1 $$\begin{aligned} \mathrm {M}^{c}_{ij}= & {} \underbrace{ \beta _0^{c} + \beta _1^{c} \mathrm {Age}_{ij}}_\text {fixed effect} + \underbrace{ b_{0i}^{c} + b_{1i}^{c} \mathrm {Age}_{ij} + \epsilon ^{c}_{ij}}_\text {random effect}, \end{aligned}$$

2 $$\begin{aligned} \mathrm {M}^{c}_{ij}= & {} \underbrace{ \beta _0^{c} + \beta _1^{c} \mathrm {Age}_{ij} + \beta _2^{c} \mathrm {Age}_{ij}^2}_\text {fixed effect} + \underbrace{ b_{0i}^{c} + b_{1i}^{c} \mathrm {Age}_{ij}+ b_{2i}^{c} \mathrm {Age}_{ij}^2 + \epsilon ^{c}_{ij}}_\text {random effect}, \end{aligned}$$

where *c* denotes the brain region or cognitive test score, $\text {M}^c_{ij}$ is the measurement of volume of region *c* or score of cognitive test *c* for subject $i = 1,\ldots ,N$ at referral $j = 1,\ldots ,n_i$. $n_i$ is the number of MRI scans or cognitive tests for subject *i*. $\text {Age}_{ij}$ is age of subject *i* at referral *j*. Age is the only predictor variable in the mixed model. The $\beta _0^{c}$, $\beta _1^{c}$, and $\beta _2^c$ are fixed effect parameters while $b_{0i}^{c}$, $b_{1i}^{c}$, and $b_{2i}^c$ are random effect parameters. $\epsilon _{ij}^{c}$ denotes the random residual errors.

**Figure 3:**
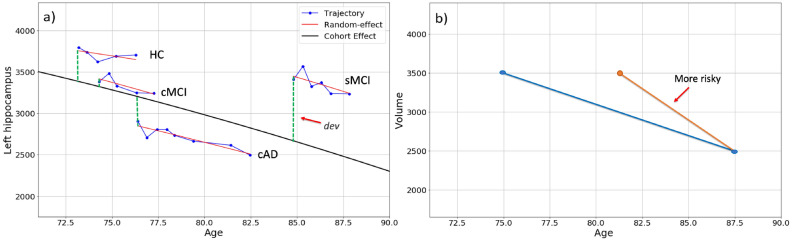
(**a**) Trajectories of age-related changes in a volumetric MRI measure (i.e., left-hippocampus) and random effects in four subjects for each of the four subgroups. The distance between the cohort effect and random effect (*dev*) of each subject (the green vertical lines) was included as one of the features in our statistical models. (**b**) The time-span was different between the participants in the present study. The change in ROI volume may therefore be the same for a participant with a short and long participation time, here illustrated by the red and blue line, respectively. The *d-slope* feature is included to capture this phenomenon^19^.

For constructing the mixed effects models we used the mixedlm function from the statsmodels Python library (v. 0.9.0). For each cognitive and MRI measure we derived the following features for each subject: (i) *r-slope*: the model-based random effects slope, thus taking the cohort effects for all subjects, and duration of study for each individual into account (the slope of the red lines in Fig. 3a). For both the linear model (Eq. 1) and the quadratic mixed models (Eq. 2), *r-slope* is $b_{1i}^c$, but for the Eq. 2 we used the coefficient of the quadratic term, $b_{2i}^c$, as an additional feature. (ii) *dev*: the distance (deviance) between the random effect line and the fixed effect line at the first time point ($\mathrm {M}_{i1} - (\beta _0 + \beta _1 \mathrm {Age}_{i1})$), thus taking the results at entry point into account (green dashed lines in Fig. 3a); (iii) *d-slope*: the slope obtained by dividing the difference of the measure at the first and last measurements by the duration between them, i.e. the slope of change from the first to the last measurement:

3 $$\begin{aligned} \mathrm {d-slope_i} = \dfrac{\mathrm {M}_{in_i} - \mathrm {M}_{i0}}{\mathrm {Age}_{in_i} - \mathrm {Age}_{i0}} \end{aligned}$$

where $\mathrm {M}_{i0}$ and $\mathrm {M}_{in_i}$ are the measurement at the first and last visits for subject *i*, respectively. This slope was used because identical changes in brain measurements or test scores can occur over different time spans, and the period of participation in the study varies for different subjects^19^ (Fig. 3b). We also added *sex* and age at last visit (*Current-Age*) before conversion, if applicable (MCI in cMCI, and AD in cAD), as features for the predictive models.

### Machine learning models and feature importance

We investigated the following experiments:

1. Classifying subjects with stable MCI (sMCI, n = 333, f/m = 143/190) vs. those who converted from MCI to AD (cAD, n = 333 , f/m = 130/203).
2. Classifying healthy controls (HC, n = 134 , f/m = 57/77) vs. those who converted from being a healthy control to MCI (cMCI, n = 134 , f/m = 55/79).

No features based on information from the point of conversion and onward were made available to the models, as they were tasked with making predictions about future diagnostic status.

In mixed effects models each group (i.e. each subject) influence the fixed effect model, and therefore impacts all the other subjects’ trajectories^27^. To avoid data leakage caused by the resulting influence on the derived features, we put aside a test set containing $20 \%$ of the subjects before creating the mixed effects models. We balanced the number of subjects in each class and controlled for gender and age. No subjects were present in both the train- and test set.

We trained an ensemble model based on a soft voting strategy, i.e. based on a weighted vote taking the models assigned probabilities into account, containing the following five models: logistic regression, support vector machine, K nearest neighbors, random forest, and a gradient boosting model. We used an ensemble approach as this tend to result in more robust classifiers that are less reliant on specific properties in the data set when compared to single classifiers^28,29^. We used confusion matrices, precision, recall and $F_1$ scores to assess our models during development and hyperparameter selection, using subject-level, leave-one-out cross-validation on the training set. For each model we set up a grid search through hyperparameters to select models that generalized well. For the logistic regression model we evaluated whether to include *l*2 penalty and the strength of regularization. For the support vector machine model we assessed various kernels (polynomial, sigmoid and radial basis function), the kernel coefficient and regularization parameter. For the K nearest neighbor model we tried multiple combinations of the number of neighbors and distance metrics. For the random forest model we searched for a good combination of the number of trees and the maximum tree depth allowed, while for the gradient boosting model we searched through both complexity and sampling parameters. To ensure fair comparison among the models trained on different sets of features, we ran new grid searches for each feature set.

To evaluate the feature importance in the classification model, we used permutation importance, also called mean decrease accuracy, as implemented in the ELI5 Python library. This is a data-driven approach to feature importance, based on measuring the decrease in model accuracy when randomly shuffling each feature separately multiple times (we used five trials for each feature). The idea is that the negative impact on performance of permuting an important feature is larger than for less important features^30^.

## Results

### Experiment 1: Prediction of sMCI vs. cAD

**Figure 4:**
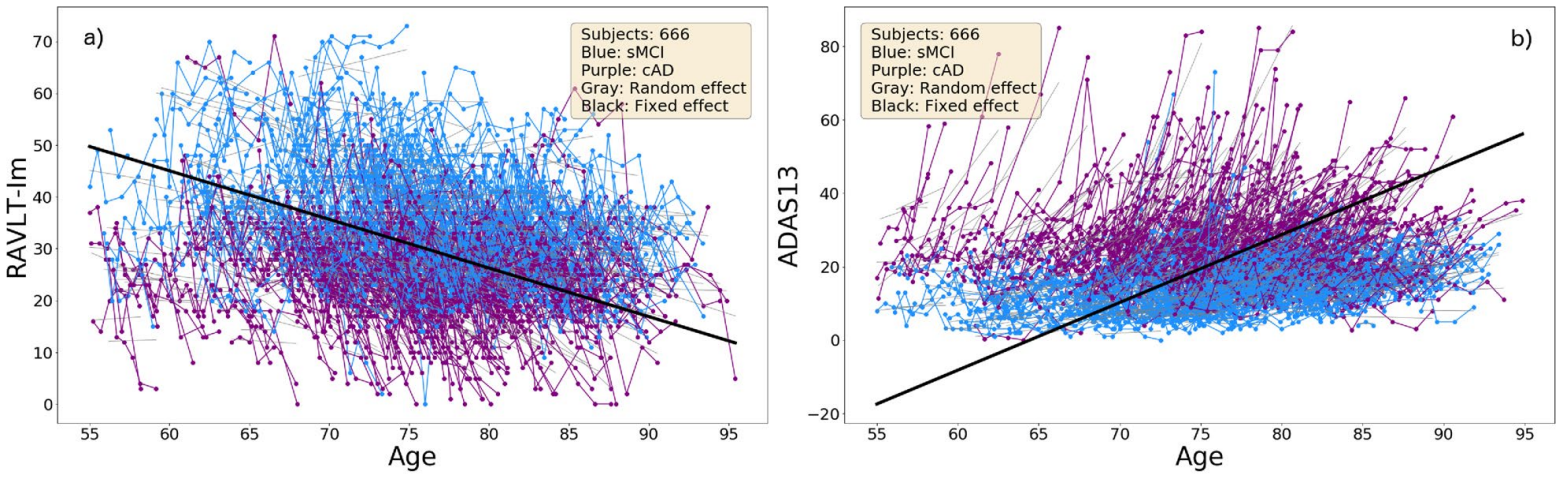
The trajectories for performances on the RAVLT-Im test (**a**) and the ADAS13 test (**b**), with age at testing on the x-axis. The thick black curve is the cohort regression line, and thin grey lines are random effects for each subject. Severity of impairment is reflected by a lower score on the RAVLT test and a higher score on the ADAS13.

The change in performances on the RAVLT-Im and ADAS13 tests are illustrated in Fig. 4. Note the age-related decline in both the sMCI and the cAD subgroups, with the most severe impairments shown within the cAD group.

**Figure 5:**
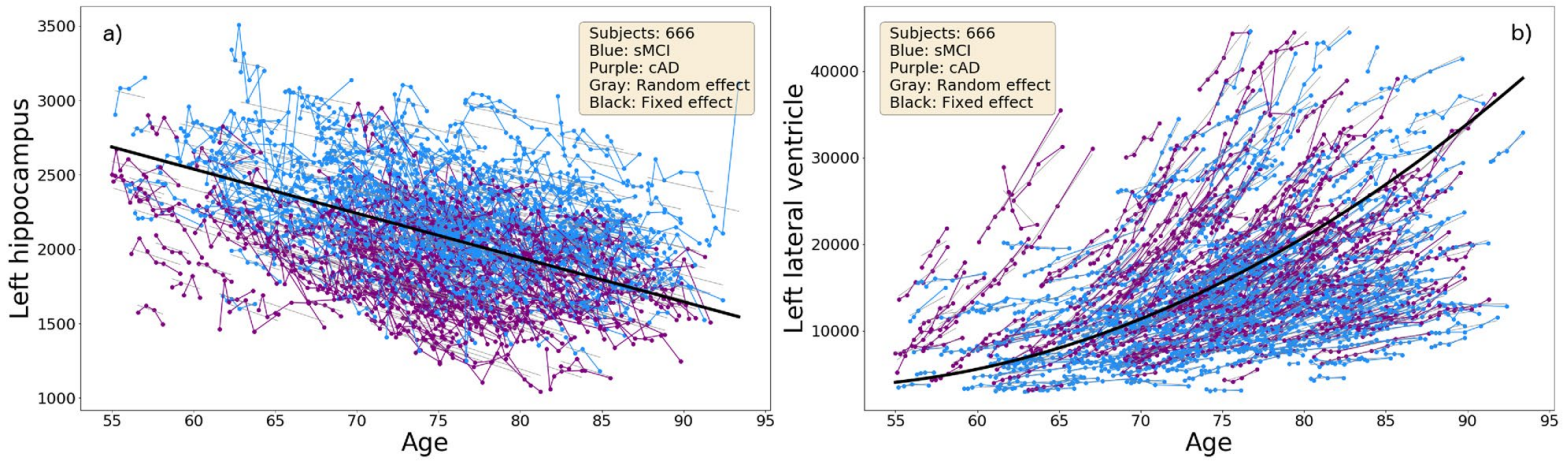
The trajectories for the normalized volumes of the hippocampus and the lateral ventricle in the left hemisphere with age at scan at the x-axis. The thick black curve is the cohort regression line, and the thin grey lines are random effects for each subject.

Figure 5 illustrates age-related tissue loss in the brain, with an almost linear shrinkage of the hippocampus volumes (Fig. 5a) and a non-linear increase in the volume of the lateral ventricle (Fig. 5b). Overall, the most extensive losses are found among subjects in the cAD subgroup.

**Figure 6:**
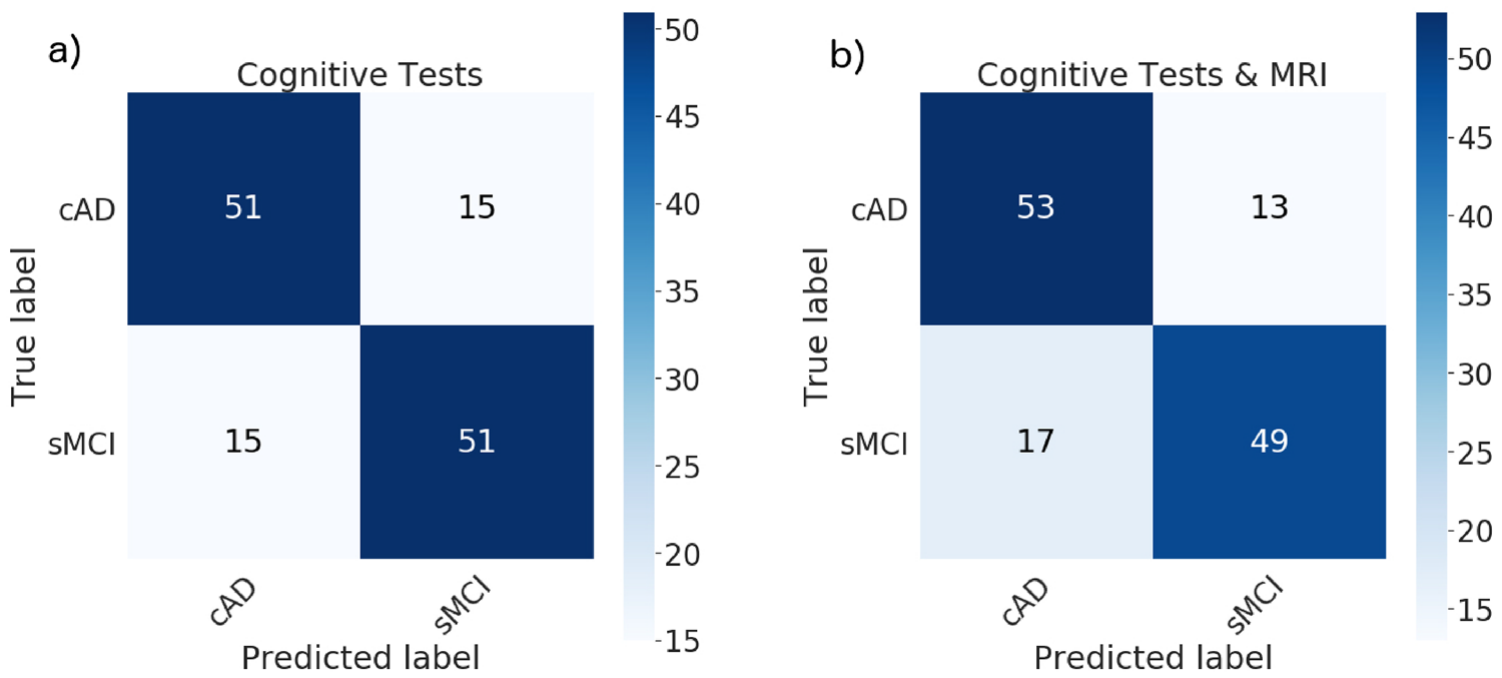
Confusion matrices for classification of sMCI vs. cAD from the cognitive features (**a**) and the combination of MRI and cognitive features (**b**).

Inclusion of the cognitive trajectory features (*r-slope*, *dev* and *d-slope* for each test measure) in the ensemble model gave $77\%$ for the accuracy, precision, recall and the $F_1$ scores. These scores changed to $77\%$, $76\%$, $80\%$ and $78\%$, respectively, when the longitudinal MRI features were added. The confusion matrices in Fig. 6 show a misclassification rate of $23\%$ for the subjects in both the cAD and the sMCI group when only the cognitive features were included, with a reduction to $20\%$ for the cAD subgroup and an increase to $26\%$ in the sMCI subgroup when the MRI features were added.

To further study these findings we performed a 15-fold cross validation experiment on the training data set, controlling for labels, age, and gender in the hold-out folds. The classifier trained on only cognitive features obtained a mean accuracy of $76\% \pm 4\%$ and the MRI features resulted in mean accuracy of $77\% \pm 3.7\%$. Note that the models tested on the original hold-out test set were optimized based on leave-one-out cross validation over the entire training data set.

**Figure 7:**
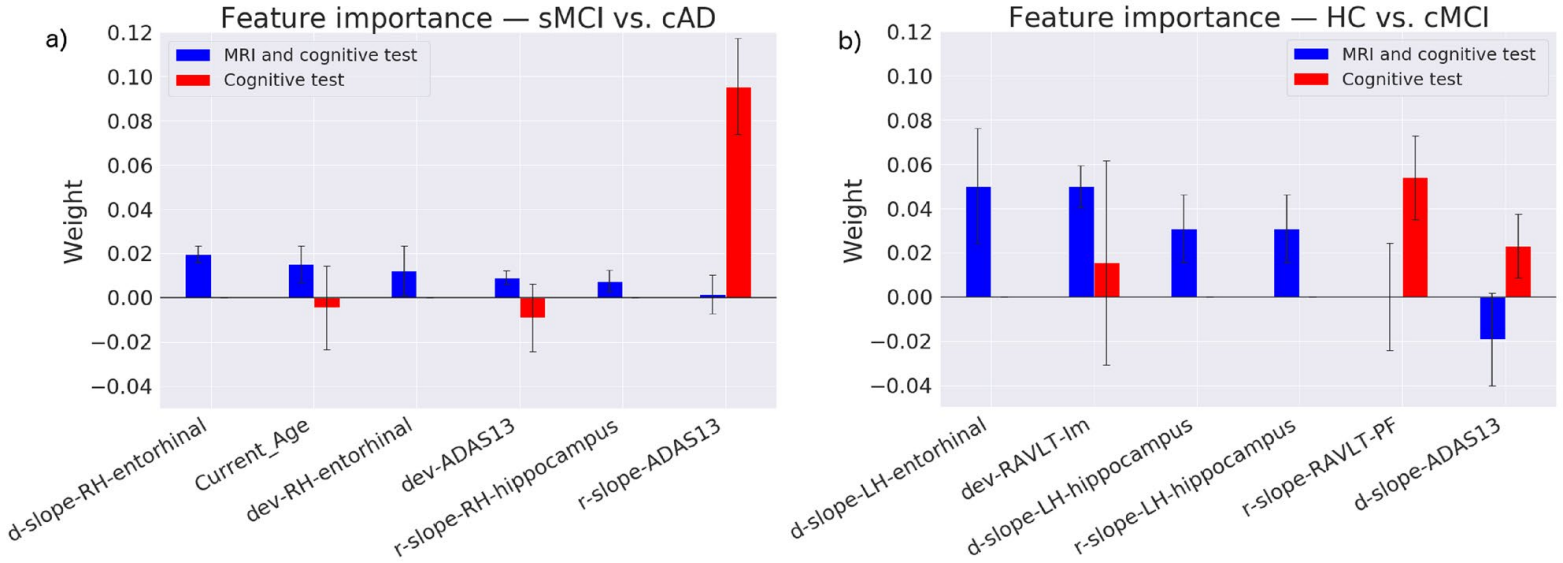
Feature weights when classifying sMCI vs. cAD (**a**) and HC vs. cMCI (**b**), based on cognitive features (in red) and the combination of MRI and cognitive features (in blue). For convenience, the plots only show a selection of the most important features after adding the MRI features to the analyses. Weights near zero and features for which the permutation importance had standard deviations greater than the estimated mean weight are not plotted. The most important features, when predicting from only the cognitive tests, were kept in the plot to illustrate the main changes observed after adding the MRI features.

The part a) of Fig. 7 shows the weights of the features in our model classifying sMCI vs. cAD. The model-based random slope (*r-slope*) of the ADAS13 trajectory provided the strongest weight among the cognitive features. When the MRI features were included in the analysis, the weight of ADAS13 decreased substantially, and became stronger for features characterising the entorhinal cortex (*d-slope* and *dev-RH entorhinal*).

### Experiment 2: Prediction of HC vs. cMCI

**Figure 8:**
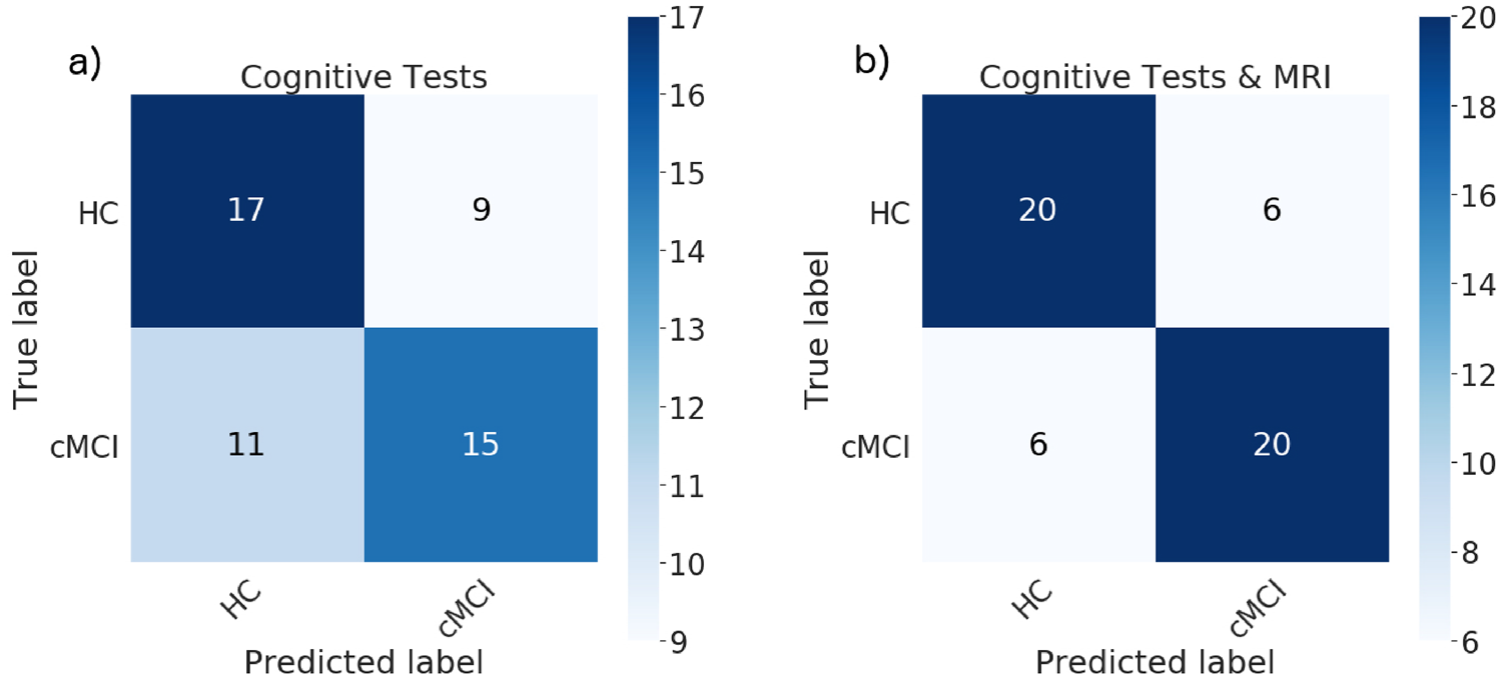
Confusion matrices for classifying HC vs. cMCI from cognitive features (**a**) and the combination of cognitive and MRI features (**b**).

With the longitudinal cognitive features as inputs to our ensemble model, we obtained an accuracy, precision, recall and $F_1$ score of $62\%$, $62\%$, $58\%$ and $60\%$, respectively. Adding the MRI features increased the accuracy, precision, recall and $F_1$ scores to $77\%$ for all. The part a) of the confusion matrix in Fig. 8 shows a somewhat lower misclassification rate for HC subjects ($35\%$) than for cMCI ($42\%$) subjects when only the cognitive features were included in the analysis. The rate decreased to $23\%$ for both subgroups when the MRI features were added (Fig. 8b).

To assess the robustness of the findings we again performed a 15-fold cross validation experiment on the training data. The classifier trained on only cognitive features gave a mean accuracy of $56\% \pm 6\%$, while the MRI features resulted in mean accuracy of $61\% \pm 5.7\%$.

The part (b) of Fig. 7 shows the feature importance for our model classifying HC vs. cMCI. The model-based random slope (*r-slope*) from a measure of memory function (RAVLT-PF) provided the strongest weight among the cognitive features. When the MRI measures were included, the *d-slope* of the entorhinal cortex in the left hemisphere and a measures of immediate memory function (*dev-RAVLT-im*) showed the strongest weights.

## Discussion

The present study used mixed effects models to define features characterising individual trajectories of change in a set of cognitive and MRI measures. These features were then used as predictors to classify subgroups with stable MCI (sMCI) vs. converters to AD (cAD) in one experiment, and to classify subgroups of healthy controls (HC) vs. converters to MCI (cMCI) in a second experiment. Visual inspections showed an age-related decline in cognitive performance and volumetric MRI measures in all subgroups. Using the features to train ensemble machine learning models gave classifications that were clearly better than chance level. For the prediction of sMCI vs. cAD, the mean classification $F_1$-score was $77\%$ when only the features characterising the trajectories of cognitive changes were included, with only one percentage point improvement when the MRI features were added. When restricted to the cognitive features, the model-based slope of the ADAS13 trajectory was given a relatively strong weight, while it was dramatically reduced and outperformed by features characterising the volume change in the entorhinal cortex when information from MRI was added. For the HC vs. cMCI predictions, the $F_1$-score was substantially improved from 60% to 77% when the MRI features were included. Among the cognitive features, a feature characterising change in memory function was given the strongest weight, followed by ADAS13. When the MRI features were added, information about the changes in the volume of the entorhinal cortex, hippocampus and the immediate memory function were given the strongest weights. The confusion matrices showed results above chance level, with the largest drop in misclassification rate when both the cognitive and MRI features were included.

The results confirmed the expected age-related change in cognitive function. Furthermore, the weight given to longitudinal features of memory function (in the HC vs. cMCI experiment) supports the sensitivity of memory tests to the early symptoms of a path leading towards a neurodegenerative disorder^10,11^, and that symptoms of an amnesic MCI may indicate a high risk of a path towards AD^6^. In a stage closer to an AD diagnosis, the results on a more global measure of cognitive function (ADAS13)^9^ are given stronger weight. Still, the contribution from MRI measures was substantial when classifying HC vs. cMCI. The design of the present study was inappropriate for identifying the exact time-point where information about MRI measures would increase the accuracy of the prediction. However, the results are still in line with studies showing that cognitive changes associated with AD tend to manifest themselves several years after the condition is well established in the brain^12^. The importance of the trajectory of change in the volume of the entorhinal cortex is also worth a comment. Entorhinal cortex acts like a relay station, with widespread connections to cortical and subcortical areas^31^. Several studies have documented that volume changes in the entorhinal cortex can be detected in an early stage of AD, and that there are strong correlations between different parts of the entorhinal cortex and memory function^32^. The present study should therefore be followed by studies on the predictive values of subcomponents of entorhinal, hippocampus and other related brain structures.

Although we obtained correct classifications above chance level, the misclassifications are too high to enable prediction on an individual level from the selected features. For converters to MCI, consideration should be given to the high number of individuals misclassified as healthy controls when the algorithms were based only on cognitive features. This illustrates the challenge in defining the fine line between healthy and pathological cognitive ageing, and the phenotypic diversity characterising the group of patients with MCI^1,2,33^. Furthermore, it may also reflect a limitation of the ADNI protocol. Although MCI is defined from the presence of subjective memory complaints, objective memory impairment, normal general cognitive function and intact activities of daily living/absence of dementia, studies have described heterogeneous subtypes, including a subgroup demonstrating intact cognitive function^34^ and MRI findings^35^. The prediction was more accurate for classification of patients converting to AD than in those with a stable MCI. This indicates the challenge in classifying an individual as AD, a diagnosis that is only definite after a post-mortem confirmation^5^. Future studies including such a definite outcome measure are therefore warranted.

The high number of participants included in the present study and the inclusion of predictive models and methods from modern machine learning frameworks^36^ are main strengths of the present study. The results in the study must, however, be interpreted in the light of several limitations. As already mentioned, this includes how we defined the subgroups. Inclusion of a small number of cognitive and MRI measures among the ones available in the ADNI dataset is another limitation. We have not provided sufficient information to specify whether the impairments in the MCI group affect single or multiple cognitive domains. And even the ADNI dataset miss out some important biomarkers^37^ and information about cognitive reserve factors (e.g.^38,39^), factors that certainly are essential to understand the phenotypic diversity of trajectories from normal function to AD. The results are also restricted by our analytic approach. The choice of models not only influence the predictive performance, but also the feature weights indicating feature importance. Furthermore, as the method used to assess feature importance is based on permuting single features, it doesn’t give a precise way to assess how combinations of features are weighed by the models. Finally, information about mean time between MRI scans and cognitive testing and number of visits, presented in Table 2, was not controlled for in the statistical models.

## Conclusion

We showed that a set of mixed effects-derived features from psychometric tests of cognitive function and an MRI examination gave predictions of healthy controls vs. MCI and stable MCI vs. AD that were above chance level. The results confirmed the importance of early changes in memory function and the role of entorhinal cortex as an imaging-based biomarker of normal and pathological ageing in older adults. Our major contributions are the application of (i) measures from the rich ADNI dataset, (ii) features defining trajectories of change in relevant cognitive and MRI measures, and (iii) a data-driven machine learning approach to assess the measures’ weights in classification models. Future studies should further investigate this avenue of brain-behaviour relationships in older age. They should consider inclusion of the wider range of genetic^40^ and environmental^41^ variables, and thus probably reduce the misclassifications shown in the present study, as well as other predictive models and methods within modern machine learning frameworks^36,42^.
